# What sports activity levels are achieved in long-term survivors with modular endoprosthetic humerus reconstruction following primary bone sarcoma resection?

**DOI:** 10.1007/s00508-020-01779-7

**Published:** 2020-12-09

**Authors:** Nikolaus W. Lang, Maximilian F. Kasparek, Lukas Synak, Wenzel Waldstein, Philipp T. Funovics, Reinhard Windhager, Gerhard M. Hobusch

**Affiliations:** Department of Orthopaedics and Traumatology, Medical University of Vienna, Vienna, Austria

**Keywords:** Bone sarcoma, Megaprosthesis, Synthetic mesh, Complications, Postoperative functional outcome

## Abstract

**Background:**

The aim of the study was to assess (1) sports activity, (2) sports involving the upper extremities, (3) functional outcome and (4) sports-related complications of long-term survivors of primary malignant bone tumors of the proximal humerus.

**Methods:**

A total of 18 patients with an endoprosthetic reconstruction for primary malignant bone sarcoma of the proximal humerus (8 male, 10 female, mean age 19.9 ± 8.4 years, range 7.8–37.4 years) with an average follow-up of 18.1 ± 7.4 years (range 6.7–29.8 years) were included. The type of sport, frequency, duration of each sport session and the University of California, Los Angeles (UCLA) activity score were assessed before surgery, at 1 year, 3 years and at the latest follow-up. Functional outcome was assessed by the Toronto extremity salvage score (TESS).

**Results:**

The mean UCLA activity score decreased from 8.0 (±1.3, range 5–9) preoperative to 4.2 (±1.7, range 3–8) at 1‑year follow-up (*p* < 0.05). After 3 years it increased to 5.1 (±1.75, range 3–8) and further to 7 (±1.8, range 4–9) at the last follow-up. The mean postoperative TESS was 80.8 (±6.4, range 75.7–91.4) at the latest follow-up. Patients who were initially more active without reconstruction including a synthetic mesh were more likely to develop soft tissue complications accompanied by proximal endoprothesis migration.

**Conclusion:**

Patients with a modular endoprosthetic reconstruction of the humerus following primary bone sarcoma resume participation in sports. Regarding the low incidence of periprosthetic infections, utilization of a synthetic mesh for reconstruction to prevent soft tissue complications in active patients should be considered.

## Introduction

The proximal humerus is the third most common site of primary bone sarcomas [1]. Endoprosthetic reconstruction is a successful treatment option for limb reconstruction after bone tumor resection or metastatic disease of the humerus [2–6]. Adequate en bloc resection of the tumor is often accompanied by pronounced loss of soft and bone tissues. Therefore, these procedures result in a loss of function of the involved extremity [7]. Moreover, chemotherapy has a negative impact on metabolic function and muscle strength, resulting in prolonged rehabilitation [8, 9]. Physical activity has been shown to reduce chemotherapy-related symptoms and to improve the sense of well-being [10]. Regular exercise also positively influences the cardiovascular system, pulmonary function and muscle strength in cancer survivors [8, 9, 11].

In general sports play an important role among the younger European population. More than 90% of the population under 30 years perform sports on a regular basis [12]; however, limited data are available about activity levels and performed form of sports of long-term survivors after primary malignant bone tumors of the proximal humerus. These data would help surgeons to counsel patients about the expected postoperative activity level. Therefore, the current study aimed to assess the following research questions: (1) what is the sport activity level in patients with modular endoprosthetic humerus reconstruction of the proximal humerus? (2) Do they perform sports involving the affected upper extremity? (3) What is the functional outcome and (4) is performing sport associated with an increased risk of complications?

## Material and methods

The study was approved by the local ethics committee (EK Nr 1466/2015) and was carried out in accordance with the Declaration of Helsinki.

All patients with a primary sarcoma of the proximal humerus and reconstruction by a modular endoprosthesis were included. The minimum follow-up for inclusion was 5 years.

A total of 106 patients were identified in the institution’s bone and soft tissue tumor registry and 45 patients had died. Of the remaining 61 patients 19 had a subsequent amputation of the affected limb due to infection, local recurrence, failure of the prosthesis or oncological complications. The average time to failure was 4.5 ± 2.3 years (range 0.9–7.2 years).

In this study 42 patients with an age between 0 and 65 years at the time of surgery were included, 22 patients were lost to follow-up and 2 patients refused to participate.

In total 18 patients (10 females, 8 males) with a mean age of 19.9 years (±8.4 years, range 7–37 years) at the time of surgery were included. The mean follow-up was 18 years (±7.8 years, range 6–26 years). All tumors were verified histologically by a specialized musculoskeletal pathology consultant. Of the patients 15 (83%) had an osteosarcoma and 3 patients (17%) a chondrosarcoma. Chemotherapy was administered according to international standardized protocols to 14 osteosarcoma patients (78%). Patients with chondrosarcoma as well as one patient with a parosteal osteosarcoma were treated by surgery alone (Table 1).

**Table 1:** Patient demographics

	Proximal humerus endoprosthesis	Total humerus endoprosthesis	Proximal humerus endoprosthesis	Total
	Osteosarcoma	Osteosarcoma	Chondrosarcoma	
*n *(%)	11 (61%)	4 (22%)	3 (17%)	18
Mean age (years)	18.4 ± 10.2	19.2 ± 5.6	27.5 ± 4.3	19.9 ± 8.4
Sex (m/f)	5/6	1/3	2/1	8/10
Op side (dominant/nondominant)	6/5	3/1	2/1	11/7
Follow-up (years)	17.4 ± 8.9	20.7 ± 4.9	18.3 ± 10.1	18.1 ± 7.8
*Chemotherapy*				
COSS 86	1	–	–	1
COSS 86c	1	1	–	2
COSS 91	–	1	–	1
COSS 96	5	2	–	7
EURAMOS	2	–	–	2
Individual	1	–	–	1
None	1	–	3	4
**Histological subtype**				
Osteosarcoma	–	1	–	1
Osteoblastic G3	2	2	–	4
Osteoblastic/chondroblastic G3	1	–	–	1
Chondroblastic G3	1	–	–	1
Anablastic/chondroblastic G3	1	–	–	1
Anablastic/osteoblastic G3	1	–	–	1
Parosteal	1	–	–	1
Teleangiectatic	2	–	–	2
Malignant fibrohistocytoma like	1	–	–	1
Nonspecific G3	2	1	–	3
Chondrosarcoma G2	–	–	3	3
**Type of endoprosthesis**				
HHMRS®	7	3	2	12
HHMRS® growth	2	–	–	2
Custom-made HHMRS®	–	1	–	1
Custom-made Salzer	2	–	1	3

*COSS 86, 86c, 91, 96* (Neo)adjuvant polychemotherapy according to the protocols of the Cooperative Osteosarcoma Study Group, *EURAMOS 1* (Neo)adjuvant polychemotherapy according to the protocols of the European and American Osteosarcoma Study Group

*HHMRS®* Howmedica Humerus Modular Replacement System

### Surgical procedures

All tumor resections were performed according to Enneking et al. [13], 8 patients (44.4%) had a Malawer type 5 [14] resection and did not have a functional deltoid muscle left postoperatively. Seven patients (38.9%) had a Malawer type 1 resection with postoperative limited axillary nerve function [14]. Three patients (14.3%) underwent deltoid muscle-sparing surgery with a functional axillary nerve.

A proximal humerus endoprosthesis was implanted in 14 patients (77.8%) and a total humerus endoprosthesis in 4 patients (22.2%). Thirteen patients received a Howmedica Humerus Modular Replacement System (HHMRS®, FA Stryker Howmedica [Kalamazoo, MI, USA]) endoprothesis and two patients received a HHMRS-expendable® endoprothesis. Three patients had reconstruction with a custom-made Salzer Endoprosthesis [15]. In eight patients, a synthetic mesh, the Ligament Advanced Reinforcement System (LARS; JK Orthomedic Ltd, Quebec, Canada) was used for soft tissue reconstruction to enhance the attachment of the remaining muscles and soft tissue.

In two further patients a single fascia lata autograft for reconstruction of the soft tissue coverage was used.

### Outcome parameters

The UCLA activity score [16], type of sports, frequency per week, and duration of each training session were assessed. Sport activities were categorized in low, medium and high-impact sports, based on the survey by Healy et al. [17], which was later modified by Mont et al. [18]. The Toronto Extremity Salvage Score (TESS) [19] was used to evaluate the functional outcome and quality of life at the latest follow-up. All data were recorded retrospectively during an interview at the latest follow-up appointment. Data were assessed for the time before diagnosis (−1), the first year after surgery (+1), 3 years after surgery (+3) and 5 years after surgery and/or time of last follow-up. In all patients, active range of motion (ROM) of the operated shoulder (abduction, flexion and external/internal rotation) was assessed. Complications were classified according to the International Society of Limb Salvage (ISOLS) classification (1. soft tissue failure, 2. aseptic loosening, 3. structural failure, 4. infection, 5. tumor progression) [20].

### Statistical analysis

Statistical analysis was performed using GraphPad Prism 6.0 (San Diego, CA, USA). The Kolmogorov-Smirnov test was used for testing of normal distribution. A two-way ANOVA and Mann-Whitney U-test were used for comparison of the UCLA activity score and hours/week of sports participation. Spearman rank correlation was used to assess correlations between different time points of the UCLA activity score and to investigate a correlation between the UCLA activity score and the TESS. A *p*-value of <0.05 was considered statistically significant.

## Results

### Activity levels

**Fig. 1:**
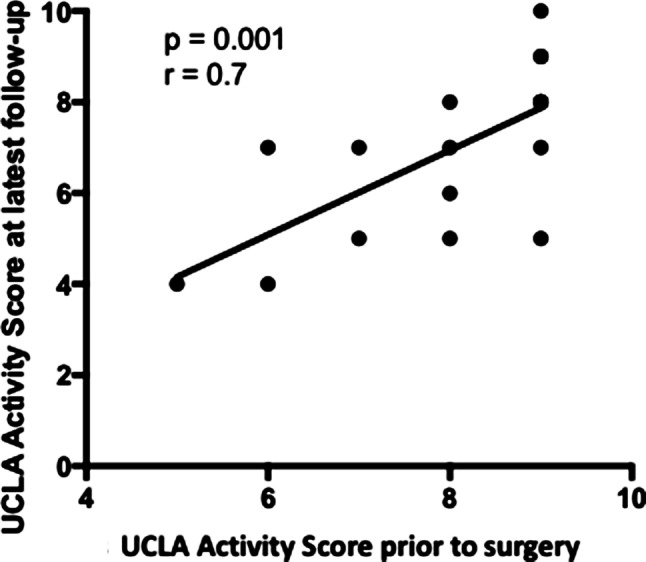
Correlation of UCLA activity score prior to surgery and at latest follow-up

The mean UCLA activity score decreased from a mean preoperative score of 8.0 (±1.3, range 5–9) to 4.2 (±1.7, range 3–8) (*p* < 0.05) 1 year postoperatively. After 3 years, the UCLA activity score increased to 5.1 (±1.75, range 3–8) and at last follow-up it further increased to 7 ± 1.8 (range 4–10) (*p* < 0.05). Patients who were more active prior to surgery, were more active at the latest follow-up (r_s_ = 0.711, *p* < 0.05), (Fig. 1).

Prior to surgery all patients (100%) were regularly participating in sports. Three patients were performing fencing, volleyball, or table tennis at tournament level. Four patients were playing soccer and one performed judo. The remaining patients were performing sports, such as swimming, jogging or training at a fitness center as recreational activities.

**Fig. 2:**
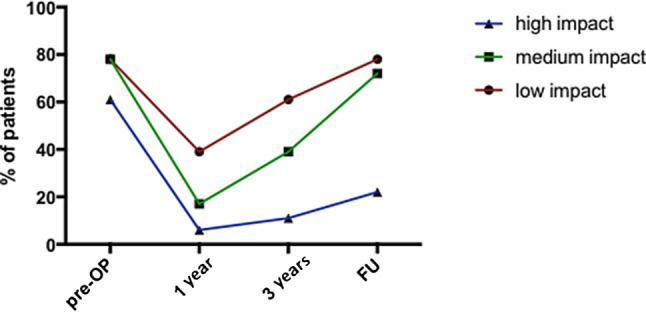
Patient participation (%) in low, moderate, and high impact sports prior to surgery (pre-OP) at 1 year, 3 years and the latest follow-up (FU) postsurgery

One year after surgery, 50% (9/18) of the patients continued to perform sports (low impact 10/18; medium impact 2/18; high impact 1/18) (*p* < 0.05). Three years after surgery, 83% (15/18) of the patients were regularly participating in sports (low impact 14/18; medium impact 2/18; high impact 2/18) (*p* < 0.05). At latest follow-up all patients (100%) resumed performing sports (*p* < 0.05) (Table 2 and Fig. 2).

**Table 2:** Sports profile of each patient within the follow-up

	Prior to surgery	+1 year postsurgery	+3 years postsurgery	Last follow-up
1	Bicycling, fitness	Nordic walking	Nordic walking	Nordic walking
2	Bicycling, fitness, skiing, swimming^a^	Bicycling, Nordic walking, skiing	Bicycling, skiing	Jogging, skiing, downhill mountain biking
3	Bicycling, fitness, swimming^a^, soccer	–	Bicycling	Bicycling, soccer
4	Bicycling, jogging, skiing, tennis^a^	–	–	Bicycling, jogging
5	Bicycling, soccer	–	Bicycling	Bicycling
6	Bicycling, Nordic walking, Nordic skiing	Bicycling, Nordic walking	Nordic walking, Bicycling	Nordic walking, bicycling
7	Fitness, judo^a^, dancing	–	Hiking, swimming^a^	Fitness, swimming^a^, dancing
–	Bicycling, volleyball^a^, hiking, swimming^a^	–	Hiking	Bicycling, fitness
9	Bicycling, fitness, swimming^a^	–	–	Hiking
10	Fitness, tennis^a^	Fitness	Fitness	Nordic walking, bicycling, fitness
11	Bicycling, fitness, hiking	Nordic walking	Nordic walking, bicycling	Nordic walking, bicycling
12	Bicycling, fitness, skiing, fencing^a^	Bicycling, Nordic walking, hiking	Nordic walking, bicycling, hiking	Bicycling
13	Hiking, skiing, tennis^a^	Skeet shooting^a^	Fitness, skeet shooting^g^	Fitness, skeet shooting^a^
14	Bicycling, fitness, soccer	–	–	Nordic walking, bicycling, fitness
15	Soccer, table tennis^a^, volleyball^a^	–	Table tennis^a^, fitness	Table tennis^a^, fitness
16	Bicycling, swimming^a^, basketball^a^	Nordic walking	Nordic walking	Nordic walking
17	Bicycling, hiking, dancing, swimming^a^	Nordic walking	Nordic walking, swimming^a^	Nordic walking, bicycling
18	Bicycling, hiking, fitness, dancing	–	Hiking	Nordic walking, bicycling, dancing

*Low impact:* swimming, hiking, Nordic walking, skeet shooting, dancing, bicycling

*Medium impact*: downhill skiing, table tennis

*High impact*: fitness, basketball, tennis, soccer, volleyball, judo, mountain biking

^a^Sports involving the upper extremities

Out of the three patients performing sports at a tournament level, one patient, who underwent tumor resection on the nondominant arm, resumed playing table tennis at a recreational level. One patient was playing soccer on weekends and one patient (tumor resection on the nondominant arm) started with downhill mountain biking 4 years after surgery and is participating in downhill mountain bike races on a regular basis. The patients who had preoperatively participated in fencing and volleyball on a tournament level, were postoperatively not able to perform these sports.

### Functional outcome

Patients changed from sports involving the upper extremities to sports involving the lower extremities. In patients with tumor resection on the dominant arm, overhead activities were not performed anymore. More than 60% (11/18) of the patients had limited ROM with an anteflexion and abduction of less than 30°.

**Fig. 3:**
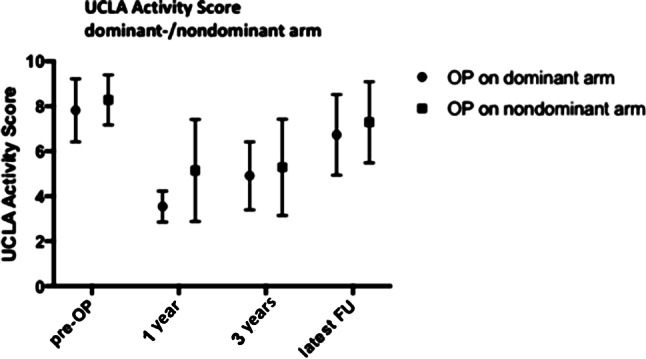
UCLA activity score; dominant vs. nondominant arm prior to surgery (pre-OP) at 1 year, 3 years and the latest follow-up (FU) postsurgery

The average TESS was 80.8 ± 6.4 (range 64.7–94.8). The UCLA activity score correlated with the TESS (*p* < 0.05, r = 0.47) indicating an influence of sports activity on general outcome following bone sarcomas. Patients with a better deltoid muscle function who were able to flex >30° in the frontal and sagittal planes had a higher UCLA activity score and TESS, respectively. Surgery on the dominant side or the nondominant arm did not influence the UCLA activity score (Fig. 3). Implantation of a proximal or total humerus endoprosthesis were not related to the postoperative UCLA activity score.

### Complications

**Fig. 4:**
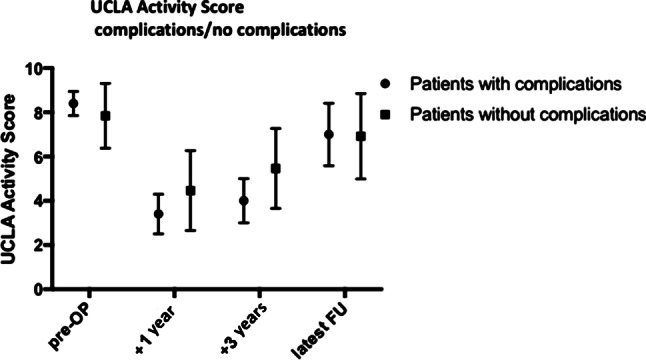
UCLA activity score; patients with complications and without complications prior to surgery (pre-OP) at 1 year, 3 years and the latest follow-up (FU) postsurgery

Nononcological complications occurred in 7 patients (39%). The most common complication was soft tissue failure with proximal endoprosthesis migration. Patients treated with a synthetic mesh showed less complications of this failure type (*p* < 0.05). The UCLA activity score was lower in patients who suffered a complication at 1‑year and 3‑year follow-up (*p* < 0.05) (Fig. 4). Higher preoperative sports levels were associated with postoperative soft tissue complications. Infections occurred in two patients treated without a synthetic mesh. Patients underwent a two-stage revision. Periprosthetic fracture, dislocation or loosening were not observed.

One patient (6%) had an oncological complication. He developed lung metastases 1.5 years after primary tumor resection, underwent metastasis resection and was tumor-free and participating regularly in sports at the last follow-up. (Table 3).

**Table 3:** Complications according to the International Society of Limb Salvage (ISOLS) classification [20]

	Type of complication					
	Type 1 Soft tissue failure	Type 2 Aseptic loosening	Type 3 Structural failure	Type 4 Infection	Type 5 Tumor progression	Total
Overall (%)	3/18 (17%) ^a^	1/18 (6%)	1/18 (6%)	2/18 (11%)	0/18	5/18 (28%)
Number of revision surgery (%)	3/7 (43%)	1/7 (14%)	1/7 (14%)	2/7 (29%)	0/7	7
Time to revision (range, years)	3.9 (0.05–6.3)	10.9	14.9	3.6 (0.7–6.4)	–	5.6 (0.05–14.9)

^a^All patients suffering from this complication were treated without a synthetic mesh

## Discussion

Limb salvage surgery is the choice of treatment of primary malignant bone tumors of the proximal humerus [21, 22]. These tumors often affect young patients and similar to same aged healthy people, sports activities plays an important role in their daily life [12, 23]. Data of the current study reveal that all patients resumed participation in sports; however, they changed to lower impact sports involving the lower extremities. These findings might help surgeons counselling patients concerning their sports expectations after limb-sparing surgery for bone sarcomas.

There are several limitations of this study: (1) we retrospectively assessed a small and heterogeneous patient population by a recall interview. (2) A total of 22 patients from abroad were lost to follow-up. (3) Patients of the current study represent a selected healthy long-term survivor collective and results might be different to patients developing oncological complications (4) Within the years and dependent on the surgeon, patients received different recommendations of sports and impact levels. This might bias performed sports as patients avoided certain activities.

Similar to prior reports [24–27] this study confirmed that physical activity of patients undergoing proximal humerus resection decreased remarkably within the first year. This might be related to the catabolic impact of major surgery accompanied by adjuvant chemotherapy; however, with increasing postoperative time, patients resumed participation in sports, and at last follow-up all patients were performing again at least a low-impact sports.

Patients who had undergone deltoid muscle-sparing surgery and partial resection tended to have a higher TESS, which might be related to the superior functional ability compared to patients requiring total deltoid resection; however, a difference in the UCLA activity score at the latest follow-up was not observed, which might be related to a general tendency to lower impact sports not involving the upper extremities. A limiting factor might be the general form of the UCLA activity score. Furthermore, the definition of low, medium and high impact activities is based on recommendations of Healy et al. and Mont et al. and was designed for patients sustaining total knee replacement and might be not appropriated for patients with primary malignant bone sarcomas of the proximal humerus [17, 18].

In comparison to tumor patients following reconstruction of the lower extremities, patients with modular endoprosthetic reconstruction of the proximal humerus had a higher UCLA activity score [26, 27]. Moreover, resection length and reconstruction with a proximal or total humerus replacement had no influence on the UCLA activity score. This might be explained by a switch from overhead activities to activities involving the lower extremities and these activities are associated with higher UCLA activity scores [16]. The main key issue for functional outcome of the affected extremity still remains the preservation of the axillary nerve [3].

The complication rate of the current study was similar to prior reports in the literature [2, 6, 28]. It was noticed that patients who were preoperatively more active, were more likely to develop soft tissue complications. Occurrence of a complication entailed a recovery of sports activity within the first 3 postoperative years. An influence of certain sports on specific complications was not noticed. Henderson et al. reported an overall infection rate in patients with proximal humerus replacement of 6.3% which is lower than in our series [20]. Our results might be influenced by the high number of patients that were lost to follow-up and those who were not included in our series.

Tang et al. reported lower soft tissue complications and better functional outcome in patients with soft tissue reconstruction using a synthetic mesh [4]. In the current series similar findings were observed as patients undergoing initial soft tissue reconstruction with a synthetic mesh had a lower rate of soft tissue complications and infection. Similarly, Henderson et al. postulated in their review that type I complications are more common in patients with shoulder or proximal femur replacement due to persisting instability [20]. This is a strong argument for the use of a synthetic mesh.

## Conclusion

Long-term survivors with an endoprosthetic reconstruction of the humerus following primary bone sarcoma resection resumed participation in sports on a regular basis. A change to lower impact sporting activities involving the lower extremities was observed. Some patients perform high-level sports, such as downhill mountain biking or soccer. Regarding the low incidence of periprosthetic infections, surgeons should consider the utilization of a synthetic mesh for reconstruction to prevent soft tissue complications, particularly in active patients.
